# TSLP is a direct trigger for T cell migration in filaggrin-deficient skin equivalents

**DOI:** 10.1038/s41598-017-00670-2

**Published:** 2017-04-04

**Authors:** Leonie Wallmeyer, Kristina Dietert, Michaela Sochorová, Achim D. Gruber, Burkhard Kleuser, Kateřina Vávrová, Sarah Hedtrich

**Affiliations:** 1 Institute for Pharmacy, Pharmacology and Toxicology, Freie Universität Berlin, Berlin, Germany; 2 Department of Veterinary Medicine, Institute of Veterinary Pathology, Freie Universität Berlin, Berlin, Germany; 3 Faculty of Pharmacy, Charles University Prague, Hradec Kralove, Czech Republic; 4 Institute of Nutritional Science, Department of Toxicology, University of Potsdam, Potsdam, Germany

## Abstract

Mutations in the gene encoding for filaggrin (*FLG*) are major predisposing factors for atopic dermatitis (AD). Besides genetic predisposition, immunological dysregulations considerably contribute to its pathophysiology. For example, thymic stromal lymphopoietin (TSLP) is highly expressed in lesional atopic skin and significantly contributes to the pathogenesis of AD by activating dendritic cells that then initiate downstream effects on, for example, T cells. However, little is known about the direct interplay between TSLP, filaggrin-deficient skin and other immune cells such as T lymphocytes. In the present study, *FLG* knockdown skin equivalents, characterised by intrinsically high TSLP levels, were exposed to activated CD4^+^ T cells. T cell exposure resulted in an inflammatory phenotype of the skin equivalents. Furthermore, a distinct shift from a Th1/Th17 to a Th2/Th22 profile was observed following exposure of T cells to filaggrin-deficient skin equivalents. Interestingly, TSLP directly stimulated T cell migration exclusively in filaggrin-deficient skin equivalents even in the absence of dendritic cells, indicating a hitherto unknown role of TSLP in the pathogenesis of AD.

## Introduction

Atopic dermatitis (AD), a chronic inflammatory skin disease, has a major detrimental impact on patient quality of life^1^. AD patients suffer from dry, red, and pruritic skin caused by a dysfunctional skin barrier and complex immune dysregulations^2, 3^. Atopic skin is characterised by enhanced epidermal proliferation, disturbed differentiation, and alterations in skin lipid composition and organisation^4, 5^. Additionally, mutations in the filaggrin (*FLG*) gene, which affect 10–50% of AD patients, contribute to functional epidermal barrier defects with subsequent allergic sensitisation^6–8^, an increased incidence of skin infection, and increased risk for several allergies and/or allergic asthma^9^. Irrespective of the *FLG* genotype, FLG expression is downregulated in AD patients, likely as a downstream effect of T helper cells type 2-derived (Th2) cytokines such as interleukin (IL-) 4 and IL-13^10^. Additionally, recent studies have demonstrated the detrimental effects of Th2 cytokines on the expression of cornified envelope proteins such as involucrin (IVL) and loricrin (LOR), tight junction proteins claudin-1 (CLDN-1) and occludin (OCLN), and anti-microbial peptides like β-defensins^11–13^.

Overall, increased levels of IL-4, IL-13, IL-25 and IL-33, as well as the keratinocyte-derived factor thymic stromal lymphopoietin (TSLP), a master regulator of Th2-driven inflammation, have been identified in the skin of AD patients, all of which are known to influence keratinocyte function and skin barrier integrity^14, 15^. Notably, several studies have linked TSLP with the development, maintenance and progression of atopic diseases including asthma and AD^16, 17^, although increased TSLP expression was observed only in skin lesions of AD patients but not in non-lesional skin or in serum samples suggesting local distribution^18, 19^. Nevertheless, TSLP was also identified as activator of sensory neurons which directly evoke itch behaviours, a further hallmark of atopic skin^20^.

TSLP is an IL-7-like cytokine that exerts its biological activities by binding to a heterodimeric receptor complex composed of the IL-7 receptor α-chain and the TSLP receptor chain^21^. This receptor complex is expressed by a wide range of immune cells including dendritic cells (DCs), macrophages and T cells^17^. Recently, TSLP receptors were found to be expressed on skin-associated T_reg_ cells mediating suppressive functions under pro-inflammatory conditions^22^. Moreover, TSLP plays an important role in, for example, the activation of DCs that subsequently prime human CD4^+^ T cells into Th2 cytokine-producing cells in local lymph nodes^19, 23, 24^. TSLP signalling in CD4^+^ T cells is also required for memory formation after Th2 sensitization^25^ and it activates group 2 innate lymphoid cells, which are further important players in the pathogenesis of multiple inflammatory skin diseases^26^.

Although the importance of TSLP in the pathogenesis of allergic diseases is widely recognised, little is currently known about the direct interplay between TSLP, filaggrin-deficient skin and naïve CD4^+^ T cells in humans. To overcome this shortcoming and investigate the effects of T cells in filaggrin-deficient skin, we report the development of an immunocompetent filaggrin-deficient skin equivalent that permits the migration of T cells into the dermis equivalent. Though *in vitro* models of inflammatory skin have previously been developed by supplementing the cell culture medium with disease associated cytokines^12, 13, 27^, these models lack actual immune cells, and thus, cannot not fully reflect the complex interplay between skin (patho) physiology and immune cells. Following successful model establishment, the regulation of cornified envelope and tight junction proteins, skin surface pH, pro-inflammatory cytokine secretion, skin lipid composition and barrier function of the skin equivalents in the presence of the T cells were assessed. Using this *in vitro* model, previously unidentified down-stream effects between filaggrin-deficient skin, TSLP expression, and T cell migration were identified.

## Results

### Exposure to CD4^+^ T cells induces inflammatory responses, increases skin surface pH and reduces skin barrier function

At day 12 of tissue cultivation, 1.5 × 10^6^ activated human CD4^+^ T cells were applied underneath the dermis equivalent, directly onto the cell culture insert membrane on which the normal (*FLG*+) and filaggrin-deficient (*FLG*−) skin equivalents are grown. The purity and activation status of the CD4^+^ T cells has been previously confirmed by flow cytometry (Supplementary Fig. S1). Histological analysis showed slight hyperproliferation and spongiosis in *FLG*− skin equivalents (with and without T cell exposure) compared to *FLG*+ skin equivalents. No major histological differences were observed between *FLG*− skin equivalents alone and after exposure to activated T cells (Supplementary Fig. S2).

**Figure 1:**
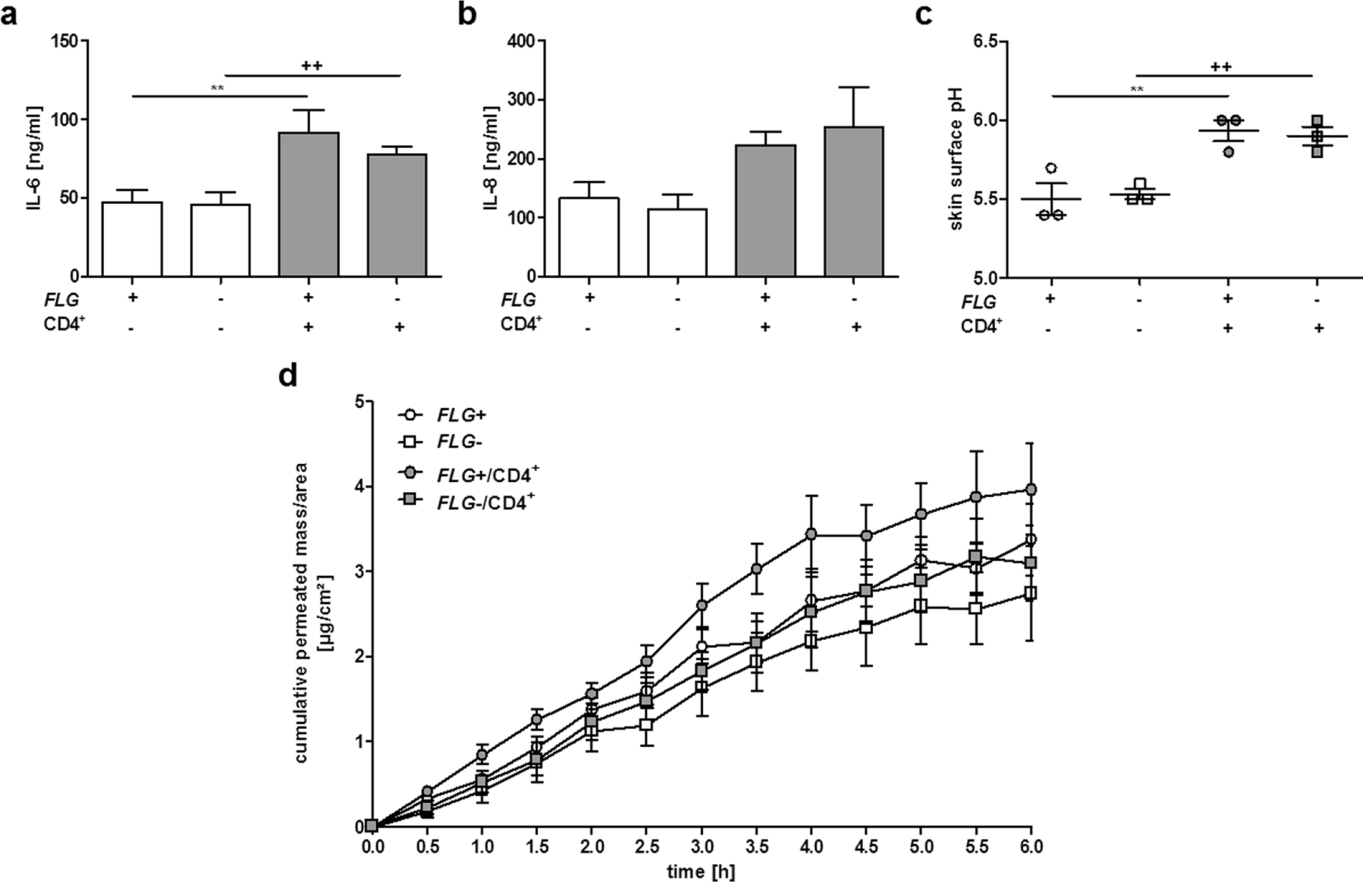
Exposure to activated CD4^+^ T cells induces inflammatory responses in normal (*FLG*+) and filaggrin-deficient (*FLG*−) skin equivalents. (**a**,**b**) Levels of the pro-inflammatory cytokines IL-6 and IL-8 in *FLG*+ and *FLG*− skin equivalents, with and without exposure to activated CD4^+^ T cells (mean ± SEM, n = 7). (**c**) Skin surface pH of *FLG*+ and *FLG*− skin equivalents before and after exposure to activated CD4^+^ T cells (mean ± SEM, n = 3). (**d**) Skin permeability of *FLG*+ (○) and *FLG*− (□) skin equivalents without activated CD4^+^ T cells and *FLG*+ (○) and *FLG*− (□) skin equivalents after exposure to activated CD4^+^ T cells (mean ± SEM, n = 4). *Indicates statistically significant differences between *FLG*+ skin equivalents (**p ≤ 0.01), ^+^indicates statistically significant differences between *FLG*− skin equivalents (^++^ *p* ≤ 0.01).

In contrast, the levels of the pro-inflammatory cytokines IL-6 and IL-8 in the culture media were significantly enhanced in *FLG*+ and *FLG*− skin equivalents following T cell exposure (Fig. 1a,b). Exposure to non-activated T cells did not increase IL-6 or IL-8 release, (Supplementary Fig. S3) and levels of IL-25 (IL-17E) and IL-33 were below detection limit. Concordant with previous results^28^, no increased skin surface pH was observed in *FLG*− compared to *FLG*+ skin equivalents (Fig. 1c). By contrast, in the presence of activated CD4^+^ T cells, skin surface pH increased significantly from pH 5.5 to pH 5.9 in both models. Additionally, the skin barrier function of *FLG*+ and *FLG*− skin equivalents was assessed by skin permeation studies using radioactively-labelled testosterone. While no distinct differences in skin permeability were observed between untreated *FLG*+ and *FLG*− skin equivalents, exposure to CD4^+^ T cells diminished the barrier function of both (Fig. 1d). P_app_ values increased from 4.1^−6^ ± 3.4^−7^ [cm/s] to 5.3^−6^ ± 3.9^−7^ [cm/s] for *FLG*+ and from 3.6^−6^ ± 4.6^−7^ [cm/s] to 4.5^−6^ ± 5.8^−7^ [cm/s] for *FLG*− skin equivalents (mean ± SEM; n = 4).

### Presence of CD4^+^ T cells reduces the expression of barrier and tight junction proteins, and disturbs their compensatory upregulation in *FLG*− skin equivalents

**Figure 2:**
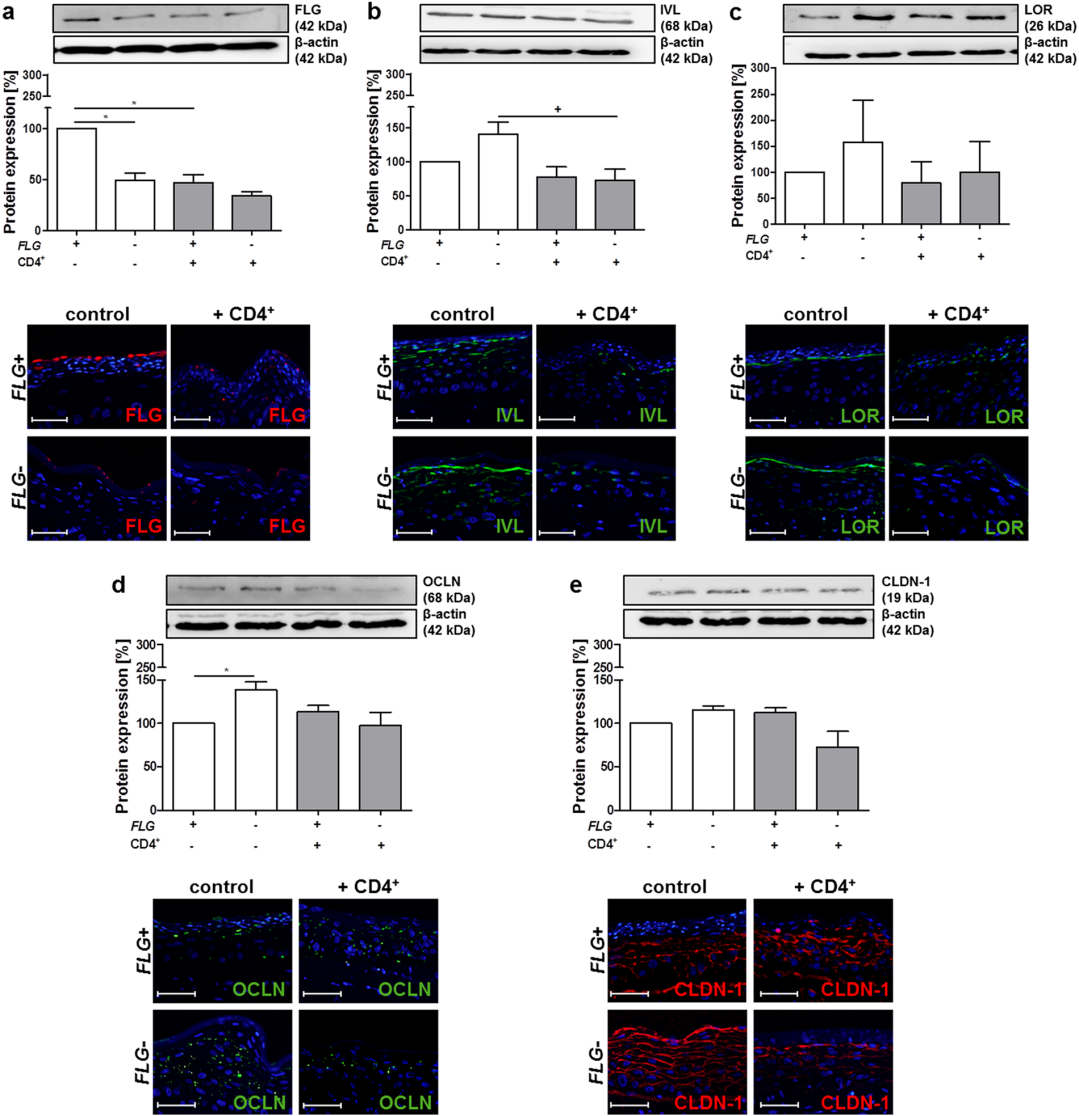
Exposure of activated CD4^+^ T cells to the skin equivalents reduces the expression of important skin barrier and tight junction proteins. Western blot and relative protein expression semi-quantified by densitometry, as well as representative immunostaining of normal (*FLG*+) and filaggrin-deficient (*FLG*−) skin equivalents alone and after addition of activated CD4^+^ T cells for (**a**) filaggrin (FLG, red), (**b**) involucrin (IVL, green), (**c**) loricrin (LOR, green), (**d**) occludin (OCLN, green) and (**e**) claudin-1 (CLDN-1, red). Counterstained with DAPI (blue), scale bar = 100 μm. *Indicates statistically significant differences between *FLG*+ skin equivalents (*p ≤ 0.05), ^+^indicates statistically significant differences between *FLG*− skin equivalents (^+^ *p* ≤ 0.05). (Mean ± SEM; n = 4).

As expected, FLG expression was significantly reduced in *FLG*− skin equivalents compared to *FLG*+ skin equivalents (Fig. 2a) as assessed by densitometry of Western blots and immunostaining. A clear trend towards upregulation of the skin barrier proteins involucrin (IVL, 1.4-fold; *p* = 0.099; Fig. 2b) and loricrin (LOR, 1.6-fold; *p* = 0.5218; Fig. 2c), as well as the tight junction proteins occludin (OCLN, 1.4-fold, Fig. 2d) and claudin-1 (CLDN-1, 1.2-fold, *p* = 0.036, Fig. 2e) in *FLG*− compared to *FLG*+ skin equivalents was observed, in line with previous reports from our group^13^. After exposure to activated CD4^+^ T cells, the compensatory up-regulation of IVL in *FLG*− skin equivalents was abolished. A similar trend, although less marked, was observed for LOR (*p* = 0.60), OCLN (*p* = 0.061) and CLDN-1 (*p* = 0.060). FLG expression in *FLG*+ skin equivalents was also diminished after T cell addition. Corresponding regulations at the mRNA level are presented in Supplementary Fig. S4.

### Exposure to CD4^+^ T cells disturbs skin lipid organisation and composition

**Figure 3:**
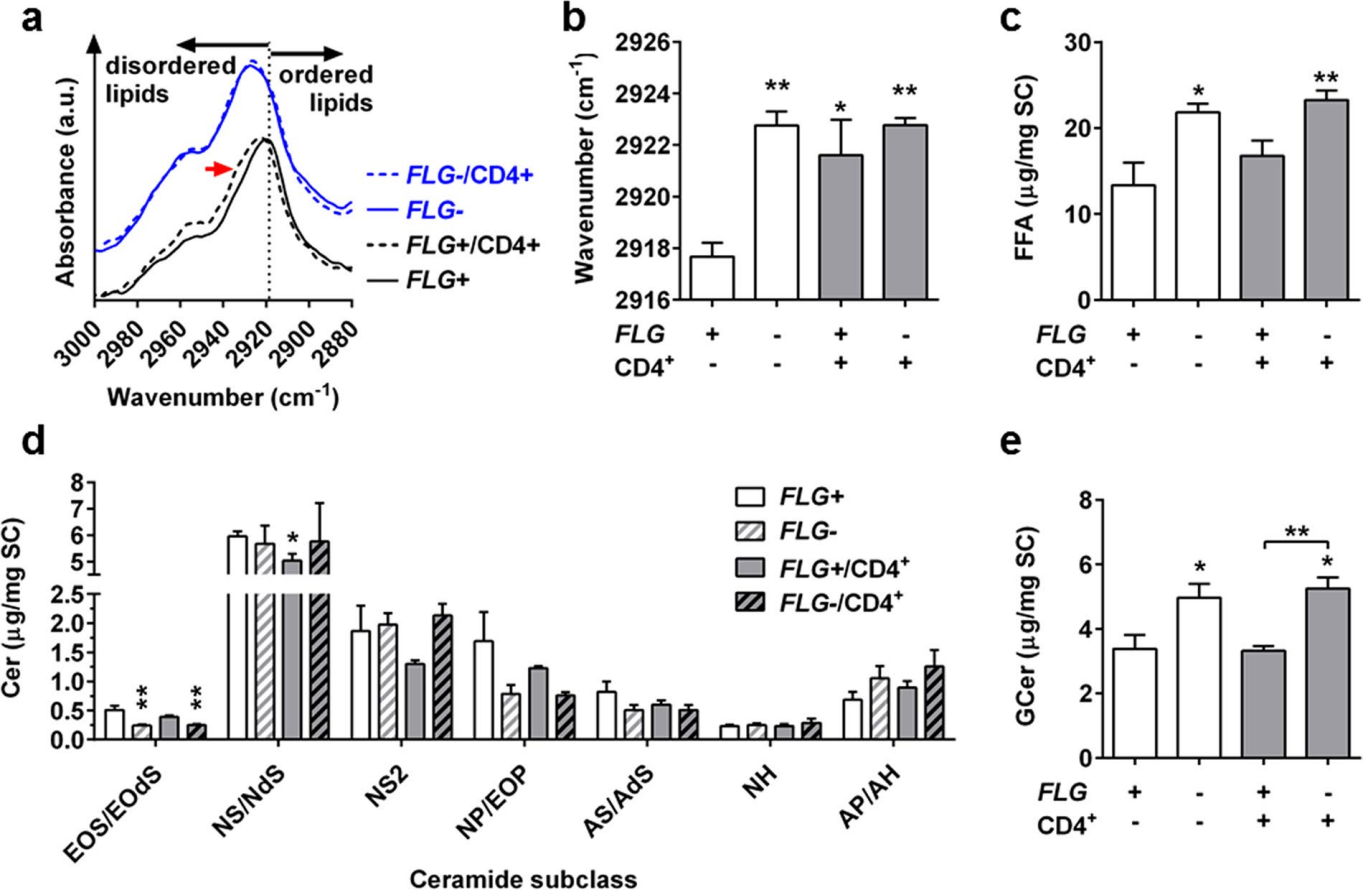
The presence of CD4^+^ T cells disorders stratum corneum (SC) barrier lipids of normal (*FLG*+), but not filaggrin-deficient (*FLG*−), skin equivalents. (**a**,**b**) Infrared spectroscopy of isolated SC (spectra and wavenumbers of methylene asymmetric stretching vibration). (Mean ± SEM; n = 4). (**c**–**e**) High-performance thin layer chromatography analysis of SC lipids (free fatty acids, ceramide subclasses and glucosylceramides) of *FLG*+ and *FLG*− skin equivalents with or without exposure to activated CD4^+^ T cells (mean ± SEM; n = 4). *Indicates statistically significant differences between *FLG*+ skin equivalents (*p ≤ 0.05, **p ≤ 0.01).

Infrared spectroscopy was used to assess the order of barrier lipids found in skin equivalents. *FLG*+ skin equivalents lipids were less ordered in the presence of activated T cells, as deduced by the increased asymmetric methylene stretching infrared vibration from 2918 cm^−1^ to >2921 cm^−1^. Diminished *FLG* levels induced similar lipid fluidity, however the presence of T cells in the *FLG*− skin constructs did not change the lipid conformation further (Fig. 3a,b).

Stratum corneum (SC) lipids of the skin equivalents were further analysed by high-performance thin layer chromatography. Both *FLG*− skin equivalents, with and without T cells, had increased levels of free fatty acids (Fig. 3c) and glucosylceramides, as compared to *FLG*+ skin equivalents without T cells (Fig. 3e). They also showed diminished levels of two sphingosine-containing ceramide subclasses, Cer EOS and Cer NS (Fig. 3d). The presence of T cells was accompanied by diminished Cer NS levels, but only in *FLG*+ skin equivalents. The levels of cholesterol, cholesteryl sulfate, sphingomyelin and phospholipids did not significantly differ between the skin equivalents (Supplementary Fig. S5).

### Distinct CD4^+^ T cell migration occurs exclusively in filaggrin-deficient skin equivalents

**Figure 4:**
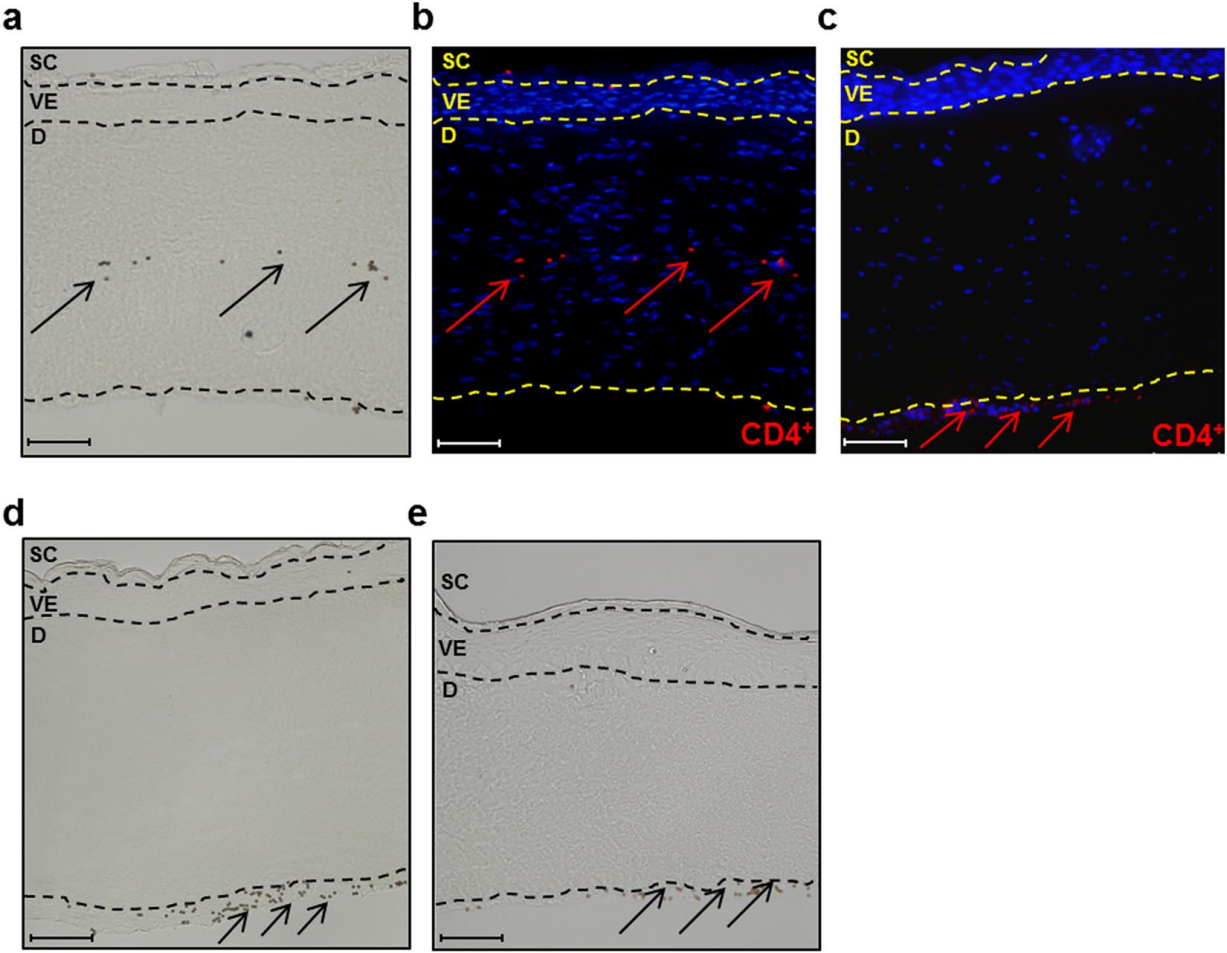
CD4^+^ T cell migration occurs exclusively in filaggrin-deficient (*FLG*−) skin equivalents. (**a**) Representative light microscopy picture of a *FLG*− skin equivalent containing migrated T cells attached to magnetic beads (arrows). (**b**) Corresponding immunostaining against CD4^+^ T cells (red), counterstained with DAPI (blue), scale bar = 100 µm. (**c**) Immunostaining against non-activated CD4^+^ T cells (red, arrows) in *FLG*− skin equivalents, counterstained with DAPI (blue). Representative light microscopy picture of (**d**) a normal (*FLG*+) skin equivalent and (**e**) *FLG*− skin equivalents generated from keratinocytes transfected with siRNA negative control after exposure to activated T cells (arrows, attached to magnetic beads; scale bar = 100 µm, SC = stratum corneum, VE = viable epidermis, D = dermis).

Unexpectedly, distinct migration of activated CD4^+^ T cells into the dermis of *FLG*− skin equivalents was observed 2 days after T cell exposure (Fig. 4a,b). In contrast, no T cell migration was observed in *FLG*+ skin equivalents, skin equivalents treated with non-activated CD4^+^ T cells, or skin equivalents generated from keratinocytes transfected with negative control siRNA (Fig. 4c–e). Additionally, T cell migration into the skin equivalents was quantified using a digital pathology scanner and corresponding software, revealing 18.95 ± 1.7 T cells/mm^2^ in *FLG*− skin equivalents and 0.9 ± 0.1 T cells/mm^2^ in *FLG*+ skin equivalents (n = 4, *p* ≤ 0.0001, mean ± SEM).

### Migration of CD4^+^ T cells in *FLG*− skin equivalents is directly stimulated by TSLP

**Figure 5:**
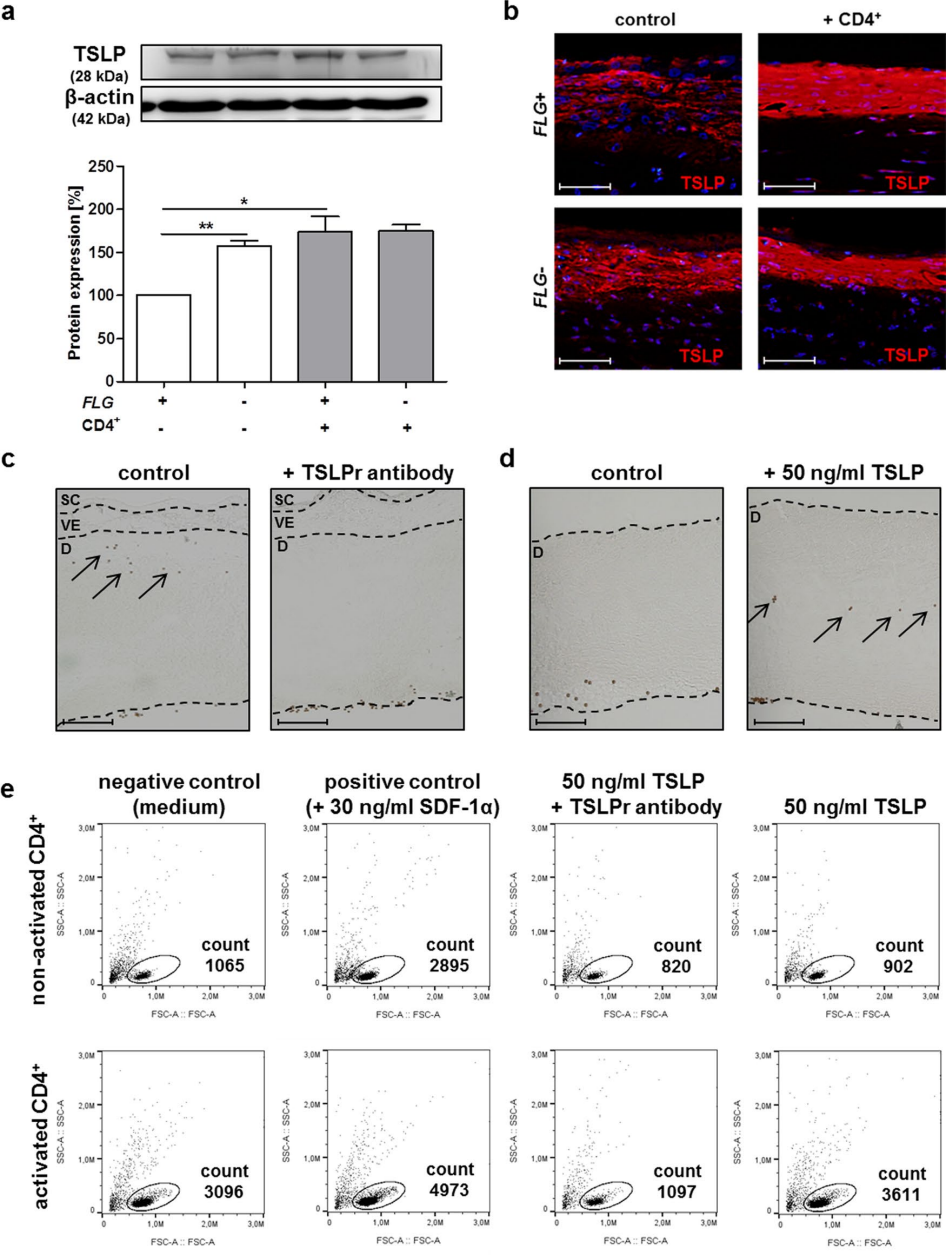
TSLP directly stimulates T cell migration in filaggrin-deficient (*FLG*−) skin equivalents. (**a**) Western blot and relative protein expression of TSLP in normal (*FLG*+) and *FLG*− skin equivalents before and after exposure to activated CD4^+^ T cells (mean ± SEM; n = 4), *indicates statistical significance over *FLG*+ skin equivalents (*p ≤ 0.05, **p ≤ 0.01). (**b**) Representative immunostains against TSLP (red) in *FLG*+ and *FLG*− skin equivalents (scale bar = 100 μm). (**c**) Representative light microscopy pictures of *FLG*− skin equivalents supplemented with activated CD4^+^ T cells (control) and with TSLPr antibody pre-incubated T cells (+TSLPr), scale bar = 100 µm. (**d**) Dermis equivalents, with and without topical application of 50 ng/ml TSLP, prior to T cell exposure (scale bar = 100 µm). (**e**) Representative dot plots from the flow cytometric transwell migration assay with non-activated and activated CD4^+^ T cells, incubated for 48 h with medium only (negative control), 30 ng/ml SDF-1α (positive control), and 50 ng/ml TSLP with and without pre-incubation with TSLPr antibody.

TSLP expression in the skin equivalents was determined by densitometry of Western blots and immunostaining. *FLG*− equivalents were characterised by intrinsically increased TSLP levels compared to normal skin equivalents (Fig. 5a), in line with previously published data from our group^13^. Here, TSLP protein expression was significantly increased by 1.6-fold relative to *FLG*+ skin equivalents (Fig. 5a,b); exposure to activated T cells did not further enhance the TSLP levels. By contrast, in *FLG*+ equivalents exposure to activated CD4^+^ T cells significantly increased the TSLP levels by 1.7-fold. Corresponding expression on mRNA level is shown in Supplementary Fig. S6.

We next investigated the role of TSLP on T cell migration in *FLG*− skin equivalents. Pre-incubation of T cells with a specific TSLP receptor antibody (2.5 µg/ml) completely abolished T cell migration in *FLG*− skin equivalents (Fig. 5c). By contrast, direct application of 50 ng/ml TSLP to keratinocyte-free dermis equivalents induced T cell migration (Fig. 5d). Transwell migration assays using 50 ng/ml also confirmed the direct stimulating effect of TSLP on T cell migration (Fig. 5e). TSLP receptor expression on the surface of CD4^+^ T cells was confirmed by flow cytometry, showing a significantly increased expression on activated compared to non-activated T cells (0.56% to 43.9%; Supplementary Fig. S7).

### TSLP shifts the polarisation profile of activated CD4^+^ T cells from Th1/Th17 to Th2/Th22

**Figure 6:**
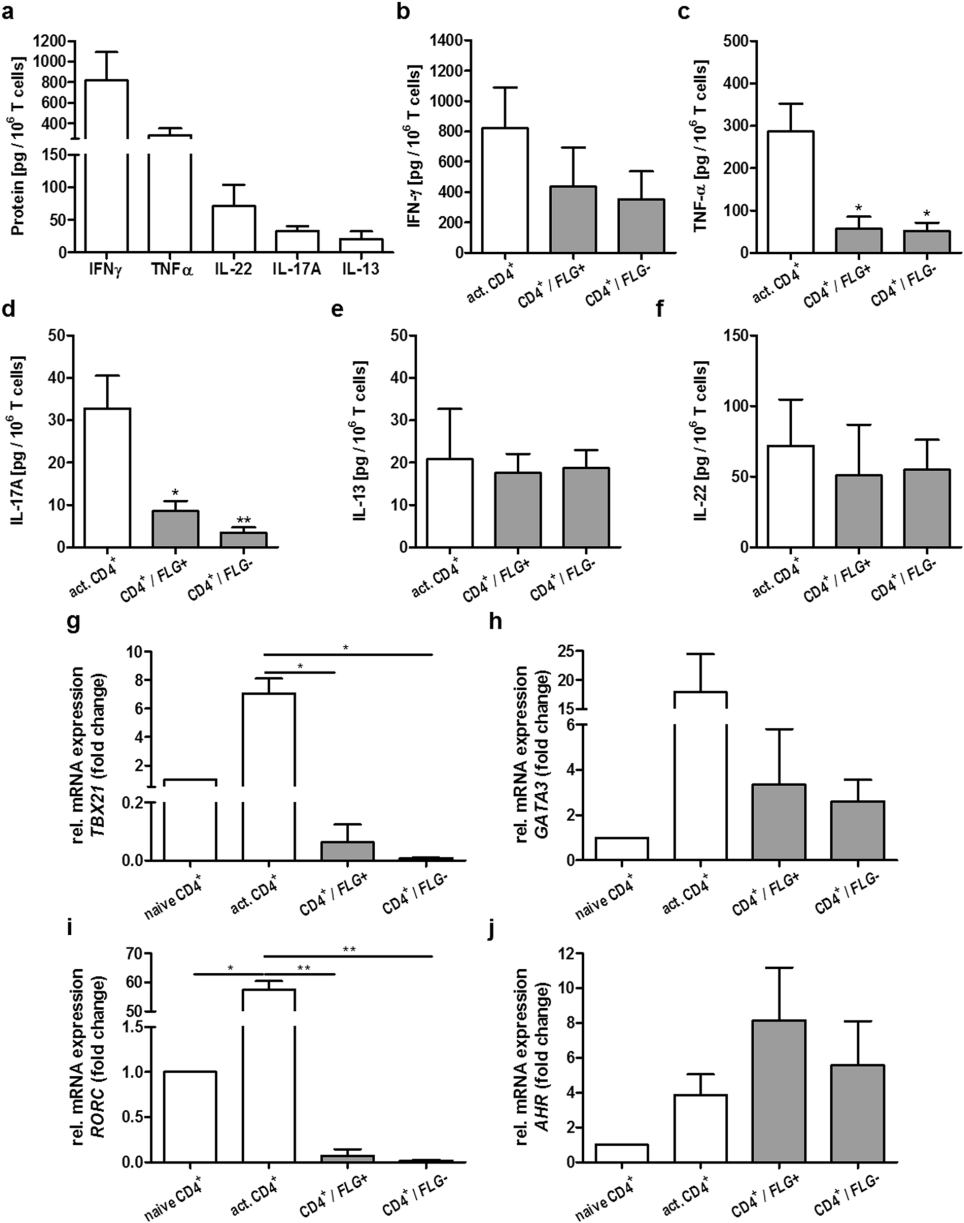
Impact of TSLP on the differentiation profile of activated CD4^+^ T cells. (**a**) Levels of IFN-γ, TNF-α, IL-22, IL-17A and IL-13 produced by activated CD4^+^ T cells (mean ± SEM; n = 3–5). (**b–f**) Respectively, release of, IFN-γ, TNF-α, IL-17A, IL-13 and IL-22 from activated CD4^+^ T cells alone (white bar) and after addition to *FLG*− skin equivalents (grey bar), relative to 10^6^ CD4^+^ T cells (mean ± SEM; n = 3–5; *p ≤ 0.05, **p ≤ 0.01). (**g–j**) Respectively, relative mRNA expression of T cell master regulators *TBX21* (Th1), *GATA3* (Th2), *RORC* (Th17) and *AHR* (Th22).

Interestingly, the enhanced TSLP levels in *FLG*+ and *FLG*− skin equivalents after exposure to the activated T cells shifted the Th1/Th17-polarisation profile of activated CD4^+^ T cells towards Th2/Th22 polarisation. Non-exposed activated CD4^+^ T cells produced high amounts of Th1/Th17 cytokines such as interferon-γ (IFN-γ), tumour necrosis factor-α (TNF-α) and IL-17A (Fig. 6a). After addition to the skin equivalents, the secretion of the Th2/Th22 cytokines IL-13 and IL-22 remained unchanged (Fig. 6e,f), but IL-17A, IFN-γ and TNF-α levels declined significantly (Fig. 6b–d). Additionally, mRNA expression analysis of the T cell master regulators *TBX21* (Th1), *GATA3* (Th2), *RORC* (Th17) and *AHR* (Th22) confirmed the shift from a Th1/Th17 to a more Th2/Th22 profile in activated T cells. Notably, this shift occurred irrespective of T cell migration into the dermis equivalents (Fig. 6g–j).

## Discussion

The crosstalk between immune and skin cells play a central role in the pathophysiology of AD and other inflammatory skin disorders^29, 30^. Nevertheless, many important pathways and correlations are still poorly understood. Generally in AD, disruption of the physical skin barrier facilitates the uptake of allergens, irritants and microbes by DCs in the skin inducing DC migration to the local lymph nodes, followed by corresponding polarisation of naïve T cells into Th2 cells^31, 32^. The exact mechanisms by which Th2 cell immunity is induced by DCs is still ambiguous, although interactions with the major histocompatibility complex-T cell receptor or the expression of OX40L are widely discussed^33^. The keratinocyte-derived cytokine TSLP was identified as a key player in the early stages of allergic inflammation, and as a sensitising product of keratinocytes released prior to the development of lesional skin in AD patients^34^. TSLP strongly activates DCs, which themselves are capable of priming naïve CD4^+^ T cells to differentiate into pro-inflammatory Th2 cells^23^. Subsequently, these allergen-specific Th2 T cells migrate back from lymph nodes to the original site of inflammation to trigger an allergic response. Pronounced dermal T cell infiltration is a hallmark of AD and may lead to chronic lesions due to continuous cytokine secretion^35^. Although only 10–50% of AD patients carry *FLG* mutations, all of them exhibit skin barrier defects due to detrimental downstream effects of pro-inflammatory cytokines on the skin barrier integrity^36–38^. Not only TSLP, IL-4 and IL-13, but also IL-17A, IL-31, IL-33 and the alarmin high-mobility group box 1 downregulate filaggrin expression and genes which are important for cellular adhesion^39–41^. Previous reports suggest that TSLP downregulates FLG expression in human skin by STAT3- and/or ERK-dependent pathways^42^.

Although the relevance of TSLP in the pathogenesis of AD is well established, little is known in humans about the direct interplay between TSLP, filaggrin-deficient skin and immune cells. Hence, to investigate these aspects in more detail, we exposed a previously developed filaggrin-deficient, human-based skin equivalent to activated human CD4^+^ T lymphocytes. To understand the effects of T cells on skin homeostasis in more detail, regulation of the skin surface pH, barrier function, cornified envelope and tight junction proteins, and skin lipid composition of the skin equivalents was assessed.

As expected, exposure to activated T cells induced an inflamed phenotype in the skin equivalents with significantly enhanced levels of pro-inflammatory cytokines IL-6 and IL-8 after exposure to activated T cells (Fig. 1a,b). Additionally, a significant increase in the surface pH of the skin equivalents (Fig. 1c) was observed, characteristic of AD patient skin lesions^43^. Interestingly, these responses occurred irrespective of T cell migration, indicating that cytokine release underneath the dermal equivalent was sufficient to induce the observed physiological aberrations. The initial compensatory upregulation of epidermal barrier (IVL, LOR) and tight junction proteins (OCLN, CLDN-1) in *FLG*− skin equivalents was abolished after exposure to the T cells (Fig. 2), well in line with recent results from IL-4/IL-13 stimulated skin equivalents^13^. The feedback mechanism is potentially disturbed by T cell-derived cytokines ultimately activating the STAT-6 signalling pathway^44^ or the S100 calcium-binding protein A11^10^. Similar compensatory mechanisms for epidermal barrier proteins have previously been reported in loricrin-deficient^45^ and flaky tail mice^46^ as well as for desmosomes and tight junction proteins^47^. Notably, here we observed a discrepancy in analysed mRNA and corresponding protein levels (Fig. 2 and Fig. S4) which is a known phenomenon and likely results from regulation controls at different expression levels. Interestingly, different studies indicate that only 40% of mRNA and protein expression correlate well^48–50^.

Exposure of *FLG*+ skin equivalents to CD4^+^ T cells also disturbed the skin lipid organisation, increased the free fatty acid content and reduced the ceramide levels in the SC (Fig. 3). Similar tendencies have previously been reported for *FLG*− skin equivalents^28, 51^. The effects on epidermal barrier proteins and skin lipids may explain the increased permeability of skin equivalents in the presence of activated T cells, although these data have to be interpreted with caution since skin equivalents show generally higher skin permeability than native human skin^52^.

Consistent with the *in vivo* situation in AD patients and previous reports from our group^13^, basal TSLP levels were significantly elevated in the *FLG*− skin equivalents (Fig. 5a,b). Additionally, TSLP levels of *FLG*+ skin equivalents also increased significantly after T cell exposure likely due to secreted cytokines. In *FLG*− skin equivalents, however, exposure to activated T cells did not further enhance the TSLP levels likely due to limited expression capacity of the soluble cytokine TSLP.

Interestingly, we observed distinct T cell migration into the dermis equivalent exclusively in *FLG*− skin equivalents (Fig. 4a,b). Since direct interactions between T cells and TSLP have recently been demonstrated^53^, we investigated the role of TSLP on T cell migration. Pre-incubation of T cells with a TSLP receptor antibody inhibited dermal infiltration, while direct application of recombinant human TSLP on the dermis equivalents or T cells cultured in transwells resulted in CD4^+^ T cell migration (Fig. 5c–e). These results clearly indicate that TSLP can directly initiate T cell migration into dermis equivalents. The lack of T cell migration in *FLG*+ skin equivalents despite enhanced TSLP levels following T cell exposure suggests a specific role of the filaggrin deficiency that requires further investigations. The migration of activated T cells due to enhanced TSLP levels was particularly unexpected, since other than that its effects on DCs, few direct biological effects of human TSLP on immune cells have been described^19, 54, 55^. Direct effects of TSLP on T cells have, however, previously been described in mice^56, 57^.

Paracrine interactions between T cells, DCs, Langerhans cells and keratinocytes play a central role in the pathophysiology of AD. DCs are known to stimulate T cell migration as well as Th2 polarisation of naïve CD4^+^ T cells^19^. Despite this, the actions of TSLP on other immune cells have been given less attention, and important pathways such as further triggers of T cell migration and the role of TSLP in CD4^+^ T cell activation/Th2 polarisation are not fully elucidated. We have now identified a previously unknown down-stream effect of TSLP in the direct activation of T cell migration. It must be noted that other potentially confounding activators of leukocyte migration, such as DCs, or chemokines involved in T cell recruitment like CCL22^58^, are absent from the skin equivalent used here. Interestingly, the enhanced TSLP levels found in the skin equivalents after T cell addition also diminished the Th1/Th17-polarisation profile of activated CD4^+^ T cells, which initially produced high amounts of Th1/Th17 cytokines IFN-γ, TNF-α and IL-17A. By contrast, secretion of Th2 cytokine IL-13 and Th22 cytokine IL-22 were unchanged (Fig. 6). This findings support the hypothesis of a decrease in Th1/Th17 profile in favour of a more Th2/Th22-like phenotype, concordant with studies suggesting that TSLP may play a prominent role in attenuating Th1 and Th17 responses^59–61^. Though a handful of studies show an increased Th17 axis in patients with AD and flaky tail mice^62–64^, overall the Th17 pathway is much less activated in patients with AD compared with psoriasis patients^65, 66^.

Despite the convincing results, a potential downside of the present study is that no information about the health status of the blood donors was provided. Hence, isolation of PBMCs from blood of AD patients or volunteers with related allergic diseases is possible, which would have affected the outcome. However, since the results are conclusive and clear, the reported data are considered as significant.

In summary, a new mechanism of T cell stimulation in human skin was identified, demonstrating the direct induction of T cell migration by TSLP in the absence of DCs. This provides a new perspective of the impact TSLP has on the pathogenesis of AD. Whilst these results will require verification *in vivo*, the skin equivalents used here allow the study of interactions between skin components that would be difficult to detect *in vivo*, due to the multitude of potentially confounding factors within this biologically complex setting.

## Materials and Methods

### Materials

All solutions for H&E staining, formaldehyde solution 4%, Tween 20 and bovine serum albumin (BSA) were obtained from Carl Roth, Karlsruhe, Germany. Horseradish-peroxidase-conjugated secondary antibodies were purchased from Cell Signaling, Frankfurt/Main, Germany. The secondary antibodies IgG DyLight 488 and IgG DyLight 594, as well as 4′,6-diamidin-2-phenylindol (DAPI) antifading mounting medium were bought from Dianova, Hamburg, Germany. The RNA isolation kit NucleoSpin^®^ RNA II was from Macherey-Nagel, Düren, Germany; the iScript^TM^ cDNA Synthesis Kit from Bio-Rad Laboratories, Munich, Germany. SYBR Green I Masterplus kit for real-time quantitative polymerase chain reaction (qPCR) was purchased from Roche, Penzberg, Germany. All primers for qPCR were purchased by TibMolbiol, Berlin, Germany.

### T cell isolation and activation

Peripheral blood mononuclear cells (PBMCs) were isolated from buffy-coat preparations from whole human blood by NycoPrep^TM^ 1.077 (Axis-Shield plc, Oslo, Norway) density gradient centrifugation. The blood was purchased from the German Red Cross (DRK-Blutspendedienst Ost, Berlin, Germany) and informed consent was obtained from the donors, respectively. The isolation of PBMCs and related experiments were performed in accordance with relevant guidelines and regulations and approved by the ethics committee of the Charité–Universitätsmedizin Berlin, Germany (EA1/227/14). Naïve human CD4^+^ T cells were purified from PBMCs by negative selection using magnetic-activated cell sorting beads according to the manufacturer’s instructions (MACS; Miltenyi-Biotec, Bergisch-Gladbach, Germany). For T cell activation, cells were stimulated with anti-CD3/CD28 beads (Life Technologies, Darmstadt, Germany), at a T cell/bead ratio of 1:1, for 24 h cultured in RPMI 1640 (Sigma-Aldrich, Munich, Germany) containing 10% heat-inactivated foetal calve serum (FCS, Biochrom, Berlin, Germany) and 2 mM L-glutamine (Sigma-Aldrich, Munich, Germany). Successful T cell/bead binding was verified by light microscopy. Thereafter, activated cells were washed and suspended in skin differentiation medium.

### Generation of skin equivalents and supplementation with activated CD4^+^ T cells

Normal (*FLG*+) and filaggrin-deficient (*FLG*−) skin equivalents were generated according to Stark *et al*.^67^. The original protocol was modified as described in previously published work^13, 28, 51^. For more details, see Supplemental Materials. All experiments were performed in accordance with relevant guidelines and regulations and were approved by the ethics committee of the Charité - Universitätsmedizin Berlin, Germany (EA1/081/13). Primary human keratinocytes and fibroblasts were isolated from juvenile foreskin obtained from circumcision surgery, informed consent was obtained, respectively. Skin equivalents generated from keratinocytes previously transfected with siRNA negative control served as control. At day 12 of tissue cultivation, 1.5 × 10^6^ activated CD4^+^ T cells were applied underneath the dermis equivalent directly onto the cell culture insert membrane of *FLG*+ and *FLG*− skin equivalents and cultured for 2 more days^68^. Skin equivalents treated with medium only served as control.

### Flow cytometry

Purity, activation status of isolated CD4^+^ T cells and expression levels of TSLP receptors on non-activated and activated T cells were analysed by flow cytometry. Cells were assessed via eight-colour flow cytometry with FACSCanto II (BD Biosciences, San Jose, CA, USA) using the antibodies depicted in Table S1. Debris was excluded by forward and side scatter gating, and dead cells by staining with Fixable Viability Dye eFluor^®^ 780 (eBioscience, Hatfield, United Kingdom). Data were analysed using FlowJo 10 software (TreeStar, Ashland, OR, USA).

### Inhibition studies

Isolated CD4^+^ T cells were pre-incubated with a TSLP receptor antibody (2.5 µg/ml; R&D Systems, Abingdon, United Kingdom) and anti-CD3/CD28 beads for 24 h. Thereafter, cells were applied under the dermis equivalent directly onto the cell culture insert membrane of *FLG*+ and *FLG*− skin equivalents. Skin equivalents supplemented with activated CD4^+^ T cells only served as positive control.

### T cell migration assay

Dermis equivalents were generated according to the normal protocol for skin equivalent generation. 24 h after airlift, a nylon mesh (200 µm; neoLab, Heidelberg, Germany) was applied on top of the dermis equivalents and 50 µl recombinant human TSLP (50 ng/ml; R&D Systems, Abingdon, United Kingdom) was applied topically over three consecutive days. Topically applied phosphate buffered saline (PBS) containing 0.1% BSA served as control. Activated T cells were applied under the dermis equivalents directly onto the cell culture insert membranes as described above.

### Transwell migration assay

For the transwell migration assay, isolated CD4^+^ T cells (activated for 3 days prior use with ImmunoCult™ Human CD3/CD28 T cell Activator; STEMCELL Technologies, Cologne, Germany) were pre-incubated with media containing 1% FCS for 4 h. 2 × 10^5^ cells/0.1 ml were applied onto the transwell membrane (5 µm pore size; Corning, Amsterdam, Netherlands). The acceptor chamber was filled with 0.3 ml cell culture media alone, supplemented with 50 ng/ml recombinant human TSLP, or supplemented with 30 ng/ml SDF-1α recombinant human CXCL12 (Miltenyi-Biotec, Bergisch-Gladbach, Germany), the latter of which served as positive control. T cells pre-incubated with a TSLP receptor antibody (2.5 µg/ml; R&D Systems, Abingdon, United Kingdom) and non-activated T cells served as negative controls. After 48 h at 37 °C and 5% CO_2_, migrated T cells were harvested and counted for 60 s at a defined constant flow rate by flow cytometry using CytoFLEX (Beckman Coulter, Krefeld, Germany). Cell debris was excluded by scatter gates.

### Histology and cell counting

For histological analysis, skin equivalents were embedded in tissue freezing medium (Leica Biosystems, Nussloch, Germany), shock-frozen with liquid nitrogen and subsequently cut into vertical sections (5 µm) with a Leica CM1510 S cryotome (Leica Biosystems, Nussloch, Germany). Sections were then stained with hematoxylin and eosin according to standard protocols.

Migrated activated CD4^+^ T cells were measured within the dermis equivalents in 4 hematoxylin and eosin-stained sections (per skin equivalent). In total, 4 different donors of *FLG*+ and *FLG*− skin equivalents were evaluated using the Aperio CS2 digital pathology scanner and the corresponding software (Table S2, Aperio nuclear V9 algorithm; Leica Biosystems, Nussloch, Germany).

### Real-time quantitative polymerase chain reaction (qPCR)

For gene expression analysis of barrier and tight junction proteins, the epidermis was gently removed, frozen and then grinded for 30 s at 25 Hz using a TissueLyzer (Qiagen, Hilden, Germany). For the analysis of T cell master regulators, the dermal compartments as well as T cells alone were lysed in lysis buffer from RNA isolation kit. RNA was isolated using the NucleoSpin^®^ RNA II kit according to the manufacturer’s instructions. For cDNA synthesis, iScript^TM^ cDNA Synthesis Kit was used. Subsequently, qPCR was performed using SYBR Green I Masterplus kit. Primer sequences are listed in Supplementary Table S3. Glyceraldehyde-3-phosphate dehydrogenase (GAPDH) served as house-keeping gene.

### Immunofluorescence and Western blot analysis

Immunofluorescence and Western blot analysis were performed according to standard protocols (see Supplementary Material). Antibodies used are depicted in Supplementary Table S4. Protein expression was semi quantified by densitometry and normalised to β-actin levels using ImageJ version 1.46r (National Institutes of Health, Bethesda, MD, USA)^69^. Potential off-target effects on respective protein expression levels due to siRNA transfection were excluded in our recent study by using keratinocytes previously transfected with siRNA negative control^13^.

### Enzyme Linked Immunosorbent Assay (ELISA)

The release of the cytokines IFN-γ, TNF-α, IL-17A, IL-22, IL-13, IL-6 and IL-8 were determined using ELISA-Ready Set Go kits (eBioscience, Hatfield, United Kingdom) according to the manufacturer’s instructions. For the analysis of IL-25 (IL-17E) and IL-33 ELISA DuoSet kits were used (R&D Systems, Abingdon, United Kingdom). Culture media of isolated CD4^+^ T cells and skin equivalents was collected and stored at −80 °C until use. To better compare the release of IFN-γ, TNF-α, IL-17A, IL-22 and IL-13 from T cells alone and after addition to skin equivalents, cytokine levels were depicted relative to 10^6^ CD4^+^ T cells.

### Skin surface pH measurements

Skin surface pH was assessed as described previously^28, 70^. For details, see Supplementary Materials.

### Skin absorption testing

Skin permeability studies were performed according to validated test procedures^71, 72^ using the radioactive labelled test compound testosterone (for details, see Supplementary Materials).

### Lipid analysis

The SC lipids were analysed in terms of their lipid content and organisation according to previously published procedures^28, 51^. For details, see Supplemental Materials.

### Statistical analysis

The unpaired student’s *t*-test was used for direct comparisons of two independent groups. For multiple comparisons, one-way analysis of variance followed by Bonferroni’s correction for multiple comparisons, was performed using GraphPad Prism 6.0 (GraphPad Software Inc., La Jolla, CA). Asterisks (*) indicate statistical significance over *FLG*+, plus (+) signs indicate statistical significance over *FLG*−. *p* ≤ 0.05 was considered statistical significant. Data from at least three independent experiments are presented as means ± standard error of the mean (SEM).

## Electronic supplementary material

Supplementary Material
