# Supplementary material for: TSLP is a direct trigger for T cell migration in filaggrin-deficient skin equivalents
Source: Sci Rep. 2017 Apr 4;7:774. doi: 10.1038/s41598-017-00670-2 (PMC5428778; doi:10.1038/s41598-017-00670-2)
Supplement: Supplementary file 1 — Supplementary Material [file 41598_2017_670_MOESM1_ESM.doc]

**TSLP is a direct trigger for T cell migration in filaggrin-deficient skin equivalents**

Leonie Wallmeyer^1^, Kristina Dietert^2^, Michaela Sochorová^3^, Achim D. Gruber^2^, Burkhard Kleuser^4^, Kateřina Vávrová^3^, Sarah Hedtrich^1*^

^1^Institute for Pharmacy, Pharmacology and Toxicology, Freie Universität Berlin, Germany

^2^Department of Veterinary Medicine, Institute of Veterinary Pathology, Freie Universität Berlin, Germany

^3^Faculty of Pharmacy, Charles University Prague, Hradec Kralove, Czech Republic

^4^Institute of Nutritional Science, Department of Toxicology, University of Potsdam, Germany

*Corresponding author: Prof. Dr. Sarah Hedtrich, Institute for Pharmacy, Pharmacology and Toxicology, Freie Universität Berlin, Germany, phone: +49 30 838 55065, fax: + 49 30 838 455065, email: [sarah.hedtrich@fu-berlin.de](mailto:sarah.hedtrich@fu-berlin.de)

**MATERIALS AND METHODS**

**Generation of skin equivalents**

Primary human keratinocytes and fibroblasts were isolated from juvenile foreskin, acquired from circumcision surgeries (with permission). To induce gene knockdown, keratinocytes were transfected (HiPerFect®; Qiagen, Hilden, Germany) with *FLG* specific siRNA (Sequence: CAGCUCCAGACAAUCAGGCACUCAU; NM_002016, Invitrogen, Darmstadt, Germany). Stealth RNAi^TM^ siRNA negative control (Med GC, sequence not shared by LifeTechnologies, Darmstadt, Germany) was used to exclude potential off-target effects.

For skin model generation, primary human fibroblasts, FCS (Biochrom, Berlin, Germany) and bovine collagen I (PureCol; Advanced BioMatrix, San Diego, CA, USA) were brought to neutral pH and poured into 3D cell culture well inserts with a growth area of 4.2 cm^2^ (BD Biosciences, Heidelberg, Germany). After 2 h at 37 °C, defined cell culture medium was added and the system transferred to an incubator with 5% CO_2_ and 95% humidity. After 2 h, primary human keratinocytes (with or without *FLG* knockdown) were added on top of the collagen matrix. After 24 h, the skin equivalents were lifted to the air-liquid interface and a differentiation medium was added. Media changes were then performed every second day.

**Immunofluorescence**

Skin sections were fixed with 4% formaldehyde, washed with PBS containing 0.0025% BSA and 0.025% Tween 20 and blocked with normal goat serum (1:20 in PBS). The sections were incubated overnight at 4 °C with primary antibodies (in PBS, 0.0025% BSA, 0.025% Tween 20; Table S4). Subsequently, the sections were incubated for additional 1 h at room temperature with secondary antibodies (1:400 in PBS, 0.0025% BSA, 0.025% Tween 20). Afterwards, the sections were embedded in 4’,6-diamidin-2-phenylindol (DAPI) antifading mounting medium and analysed with fluorescence microscopy (BZ-8000; Keyence, Neu-Isenburg, Germany). Exposure times: TSLP - red channel 1/8 s; FLG - red channel 1/10 s; IVL - green channel 1/12 s; LOR - green channel 1/13 s; OCLN - green channel 1/10 s; CLDN-1 - red channel 1/10 s; DAPI - blue channel 1/55 s.

**Western blot**

The epidermis was gently removed and subsequently lysed in radioimmunoprecipitation assay buffer. Total protein concentrations were determined using the Pierce® BCA Protein Assay Kit (Thermo Scientific, Schwerte, Germany). Subsequently, samples (~30 μg protein) were boiled in standard SDS-PAGE sample buffer and separated by 10% SDS polyacrylamide gel electrophoresis (Bio-Rad, Munich, Germany). Gels were blotted onto nitrocellulose membranes (Bio-Rad, Munich, Germany). After blocking with 5% skimmed-milk powder for 1 h at 37 °C, membranes were incubated with primary antibodies at 4 °C overnight (Table S4). Blots were washed and incubated with anti-rabbit or anti-mouse horseradish-peroxidase-conjugated secondary antibody (Cell Signaling, Frankfurt/Main, Germany) for 1 h. Afterwards, blots were developed with SignalFire™ ECL reagent (Cell Signaling, Frankfurt/Main, Germany) and visualised by a PXi/PXi Touch gel imaging system (Syngene, Cambridge, UK).

**Skin surface pH measurements**

For skin surface pH measurement, optical sensor foils for pH-imaging containing pH-indicator microparticles (fluorescein isothiocyanate) and reference microparticles (ruthenium(II)-tris(4,7-diphenyl-1,10-phenanthroline)) were applied onto the skin equivalents as described previously^1,2^. After equilibration, a RGB image was recorded using the VisiSens system for 2D pH-imaging (Presens, Regensburg, Germany) and calculations were done with the corresponding VisiSens AnalytiCal 2 software.

**Skin absorption testing**

Skin permeability studies were performed according to validated test procedures^3,4^. A testosterone stock solution (40 µg/ml, 2% [v/v] Igepal® CA-630, Sigma-Aldrich, Munich, Germany) was spiked with an appropriate amount of 2,4,6,7‑^3^H‑testosterone (100 Ci/mmol, Amersham, Freiburg, Germany) to achieve a total radioactivity of 2 µCi/ml. Permeation experiments were performed using Franz diffusion cells (PermeGear, Hellertown, PA, USA). The total amount of permeated testosterone was quantified using radiochemical detection (Microbeta Plus, Wallac, Turku, Finland). The permeation rate for testosterone was calculated as the apparent permeability coefficient (P_app_).

**Stratum corneum (SC) isolation**

The skin equivalents were placed on a filter paper soaked with 0.25% trypsin in PBS. The isolated SC sheets were washed with PBS and any remaining keratinocytes were removed with a cotton swab. Subsequently, SC sheets were washed with acetone to remove surface contaminants, vacuum-dried, aerated with nitrogen to avoid oxidative processes and stored at -20 °C. Isolated human SC served as control.

**FTIR spectroscopy**

IR spectra of the samples were collected on a Nicolet 6700 FTIR spectrometer (Thermo Scientific, Waltham, MA, USA) equipped with a single-reflection MIRacle attenuated total reflectance (ATR) germanium crystal at 23 °C. The spectra were generated by co-addition of 256 scans collected at 2 cm‑1 resolution and analysed with the Bruker OPUS software (Bruker Corp, Billerica, MA, USA).

**Isolation of stratum corneum (SC) lipids**

SC lipids were extracted with 1 ml CHCl_3_/MeOH 2:1 (v/v) per mg of SC for 2 h followed by 0.5 ml of the same solvent for 1 h. Extracted solutions were combined and concentrated under a stream of nitrogen. The lipids were dried and stored at -20 °C under argon.

**HPTLC lipid analysis**

Lipid analysis was performed on silica gel 60 HPTLC plates (20 × 10 cm, Merck, Darmstadt, Germany) as previously described^5^. Lipids for standard curves were either purchased (Avanti Polar Lipids, Alabaster, AL, USA) or synthesised^6^. To generate calibration curves, lipids were mixed in ratios that approximately correspond to the composition of human SC^7^.

**REFERENCES**

1 Schreml, S. *et al.* Luminescent dual sensors reveal extracellular pH-gradients and hypoxia on chronic wounds that disrupt epidermal repair. *Theranostics.* **4**, 721-735 (2014).

2 Vávrová, K. *et al.* Filaggrin deficiency leads to impaired lipid profile and altered acidification pathways in a 3D skin construct. *J Invest Dermatol.* **134**, 746-753 (2014).

3 Schäfer-Korting, M. *et al.* The use of reconstructed human epidermis for skin absorption testing: Results of the validation study. *Altern Lab Anim.* **36**, 161-187 (2008).

4 OECD, T. G. 428: Skin absorption: in vitro Method. *OECD Guidelines for the Testing of Chemicals, Section.* **4** (2004).

5 Wallmeyer, L. *et al.* Stimulation of PPARalpha normalizes the skin lipid ratio and improves the skin barrier of normal and filaggrin deficient reconstructed skin. *J Dermatol Sci.* **80**, 102-110 (2015).

6 Opálka, L. *et al.* Scalable Synthesis of Human Ultralong Chain Ceramides. *Org Lett.* **17**, 5456-5459 (2015).

7 Pullmannová, P. *et al.* Effects of sphingomyelin/ceramide ratio on the permeability and microstructure of model stratum corneum lipid membranes. *Biochim Biophys Acta.* **1838**, 2115-2126 (2014).

**TABLES**

Table S1. Antibodies used for flow cytometry.

| **Antibody** | **Dye** | **Clone** | **Concentration** | **Company** |
| --- | --- | --- | --- | --- |
| CD4 | PerCp | SK3 | 1:20 | BioLegend, San Diego, CA, USA |
| CD25 | APC | BC96 | 1:20 | eBioscience, Hatfield, United Kingdom |
| CD45RA | eF450 | HI100 | 1:20 | eBioscience, Hatfield, United Kingdom |
| CD45RO | PE-Cy7 | UCHL1 | 1:50 | eBioscience, Hatfield, United Kingdom |
| CD8 | FITC | 145-2C11 | 1:200 | Deutsches Rheuma-Forschungszentrum Berlin, Germany |
| TSLPR | PE | 1B4 | 1:20 | BioLegend, San Diego, CA, USA |
| CD14 | PE-Cy7 | M5E2 | 1:20 | BD Biosciences, San Jose, CA, USA |
| CD19 | FITC | HIB19 | 1:20 | eBioscience, Hatfield, United Kingdom |
| CD56 | PE | CMSSB | 1:20 | eBioscience, Hatfield, United Kingdom |
| CD154 | VioBlue | REA238 | 1:10 | Miltenyi-Biotec, Bergisch-Gladbach, Germany |
| CD15 | APC | VIMC6 | 1:11 | Miltenyi-Biotec, Bergisch-Gladbach, Germany |
| HLA-DR | APC-Cy7 | R30 | 1:200 | Deutsches Rheuma-Forschungszentrum Berlin, Germany |

**Table S2. Algorithm nuclear v9 used for cell counting.**

| **Stain 1** |  |  |
| --- | --- | --- |
|  | S1 Number of Visible Stains | 2 |
|  | S1 Target | Biomarker 1 |
|  | S1 Colour | Lock |
|  | S1 Values | Show |
|  | -- Stain-1 (Red) | 0.142 |
|  | -- Stain-1 (Green) | 0.798 |
|  | -- Stain-1 (Blue) | 0.318 |
| **Stain 2** |  |  |
|  | S2 Number of Visible Stains | 2 |
|  | S2 Target | Counterstain |
|  | S2 Colour | Lock |
|  | S2 Values | Hide |
| **Nuclei Identification** |  |  |
|  | Type | Default |
|  | Method | Automatic |
|  | -- Threshold Lower Limit | 0 |
|  | -- Threshold Upper Limit | 190 |
|  | Smoothing (um) | 2 |
|  | Merging | 0.4 |
|  | Trimming | Medium |
| **Nuclei Exclusion** |  |  |
|  | Min Size (um^2) | 4 |
|  | Max Size (um^2) | 100 |
|  | Roundness | 0.4 |
|  | Compactness | 0 |
|  | Elongation | 0 |
|  | Remove Light Objects | 0.3 |
| **Scoring Criteria** |  |  |
|  | Cytoplasmic Correction | 230 |
|  | Weak(1+) Threshold | 210 |
|  | Moderate(2+) Threshold | 188 |
|  | Strong(3+) Threshold | 162 |
|  | Dark Nuclei Removal | 0 |
| **Advanced** |  |  |
|  | Image Zoom | 0.987 |
|  | Markup Compression Type | Same as processed image |
|  | Compression Quality | 30 |
|  | Classifier Neighbourhood | 0 |
|  | Classifier | None |
|  | Class List |  |
|  | Clear Area Intensity | 240 |

**Table S3. Primer sequences for qPCR.**

| **Gene** | **Primer sense 5’-3’** | **Primer antisense 5’-3’** |
| --- | --- | --- |
| *GAPDH* | *CTCTCTGCTCCTCCTGTTCGAC* | *TGAGCGATGTGGCTCGGCT* |
| *FLG* | *AAGGAACTTCTGGAAAAGGAATTTC* | *TTGTGGTCTATATCCAAGTGATCCAT* |
| *IVL* | *TCCTCCAGTCAATACCCATCAG* | *CAGCAGTCATGTGCTTTTCCT* |
| *LOR* | *TCATGATGCTACCCGAGGTTTG* | *CAGAACTAGATGCAGCCGGAGA* |
| *CLDN-1* | *GCGCGATATTTCTTCTTGCAGG* | *TTCGTACCTGGCATTGACTGG* |
| *OCLN* | *TGCATGTTCGACCAATGC* | *AAGCCACTTCCTCCATAAGG* |
| *TSLP* | *CCCAGGCTATTCGGAAACTCAG* | *CGCCACAATCCTTGTAATTGTG* |
| *TBX21* | TTGAGGTGAACGACGGAGAG | CCAAGGAATTGACAGTTGGGT |
| *GATA3* | GAACCGGCCCCTCATTAAG | ATTTTTCGGTTTCTGGTCTGGAT |
| *RORC* | CAATGGAAGTGGTGCTGGTTAG | GGGAGTGGGAGAAGTCAAAGAT |
| *AHR* | CAAATCCTTCCAAGCGGCATA | CGCTGAGCCTAAGAACTGAAAG |

**Table S4**. Antibody dilutions for immunofluorescence (IF) and Western blot (WB).

| **Antibody** | **Isotype** | **Clone** | **IF** | **WB** | **Company** |
| --- | --- | --- | --- | --- | --- |
| CD4 | mouse IgG1 | monoclonal (4B12) | 1:50 | - | DAKO, Hamburg, Germany |
| FLG | rabbit IgG | polyclonal | 1:1000 | 1:1000 | Abcam, Cambridge, United Kingdom |
| IVL | rabbit IgG | polyclonal | 1:1000 | 1:1000 | Abcam, Cambridge, United Kingdom |
| LOR | rabbit IgG | polyclonal | 1:500 | 1:1000 | Life Technologies, Darmstadt, Germany |
| TSLP | rabbit IgG | polyclonal | 1:1000 | 1:1000 | Abcam, Cambridge, United Kingdom |
| CLDN-1 | mouse IgG2 | monoclonal (1C5-D9) | 1:300 | 1:500 | Novus Biologicals Cambridge, United Kingdom |
| OCLN | mouse IgG1 | monoclonal (1G7) | 1:300 | 1:500 | Novus Biologicals Cambridge, United Kingdom |
| β-actin | mouse IgG1 | monoclonal (15G5A11/E2) | - | 1:10000 | Sigma-Aldrich, Munich, Germany |

**FIGURES**


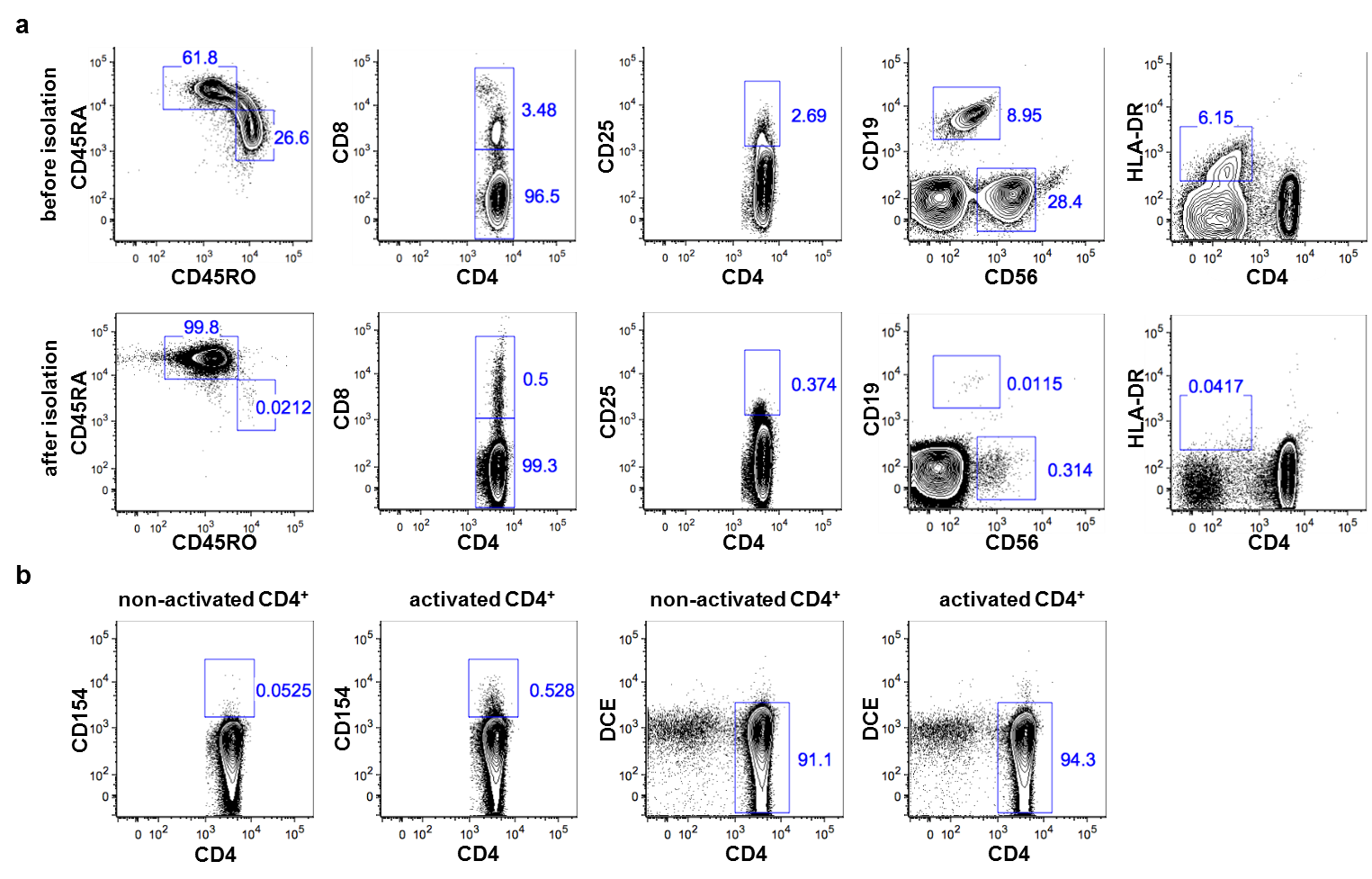


**Figure S1.** (**a**) Purity of CD4^+^ T cell isolation was examined using eight-colour flow cytometry. Surface marker expression of CD45RO (memory T cell marker), CD45RA (naïve T cell marker), CD8 (cytotoxic T cell marker), CD25 (regulatory T cell marker), CD56 (natural killer cell marker), CD19 (B cell marker) and HLA‑DR (monocyte cell marker) was analysed before and after isolation of CD4^+^ T cells from peripheral blood mononuclear cells (PBMCs) to verify the purity of CD4^+^ cells. (**b**) Flow cytometry analysis of the early T cell activation marker CD154 (CD40L) and dead cell exclusion (DCE) staining on CD4^+^ T cells before and after activation with anti-CD3/CD28 for 6 h. After 6 h the amount of activated T cells increased tenfold and 94.3% of T cells were still viable.


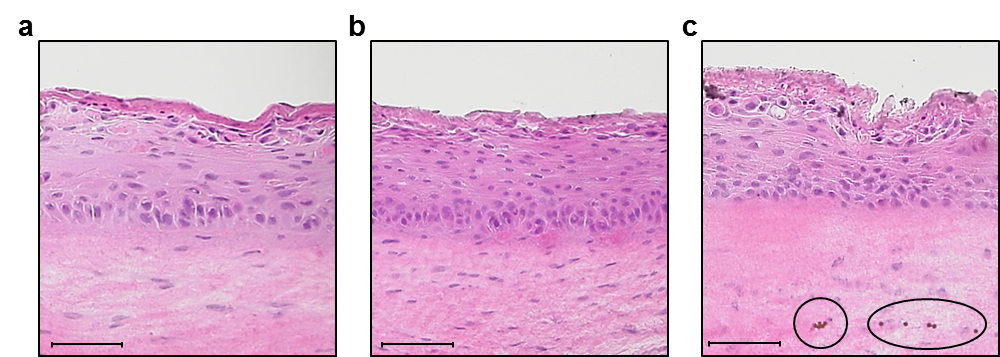


**Figure S2.** Representative hematoxylin & eosin staining of (**a**) a normal skin equivalent, (**b**) a filaggrin-deficient (*FLG*-) skin equivalent and (**c**) a *FLG*- skin equivalent containing migrated T cells attached to magnetic beads (circles), scale bar = 100 μm.


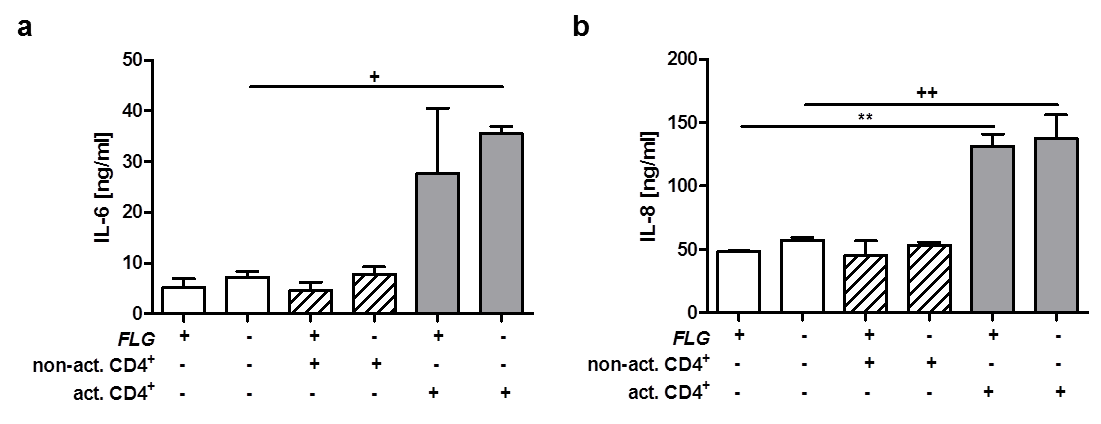


**Figure S3**. Levels of the pro-inflammatory cytokines (**a**) IL‑6 and (**b**) IL‑8 in *FLG*+ and *FLG*- skin equivalents alone and after exposure to activated or non-activated CD4^+^ T cells, respectively (mean ± SEM, n = 2). * Indicates statistically significant differences from *FLG*+ skin equivalents (***p* ≤ 0.01), + indicates statistically significant differences from *FLG*- skin equivalents (+*p* ≤ 0.05, ++*p* ≤ 0.01).


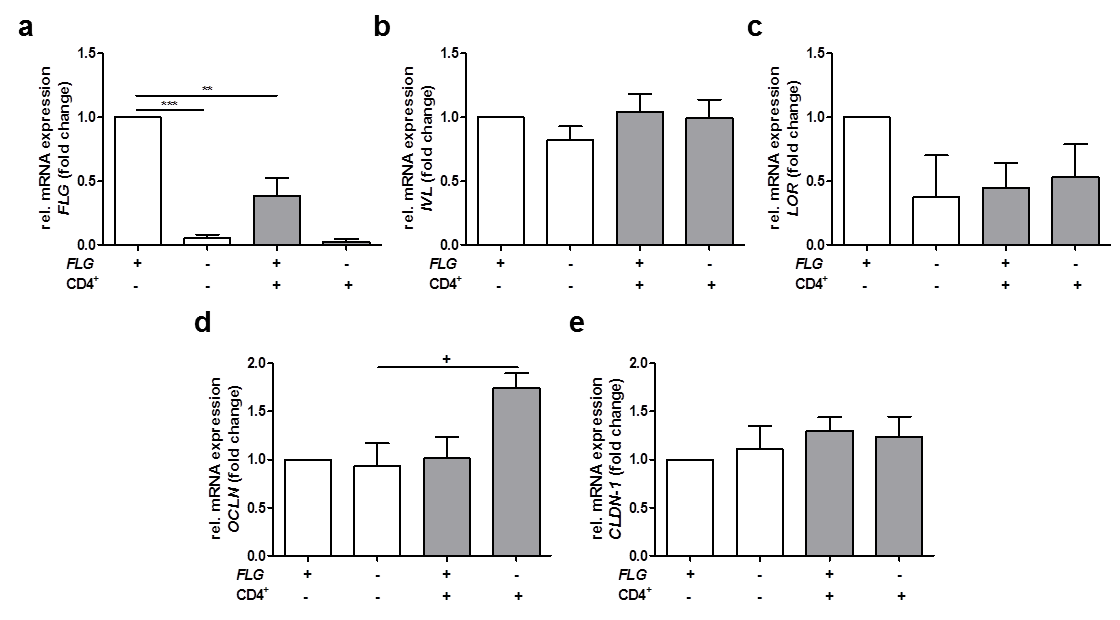


**Figure S4.** Relative mRNA expression of (**a**) filaggrin (*FLG*), (**b**) involucrin (*IVL*), (**c**) loricrin (*LOR*), (**d**) occluding (*OCLN*) and (**e**) claudine‑1 (*CLDN-1*) in normal (*FLG*+) and filaggrin-deficient (*FLG*-) skin equivalents alone and after addition of activated CD4^+^ T cells (mean ± SEM, n = 4), * indicates statistically significant differences between *FLG*+ skin equivalents (***p* ≤ 0.01, ****p* ≤ 0.001), + indicates statistically significant differences between *FLG*- skin equivalents (+*p* ≤ 0.05).


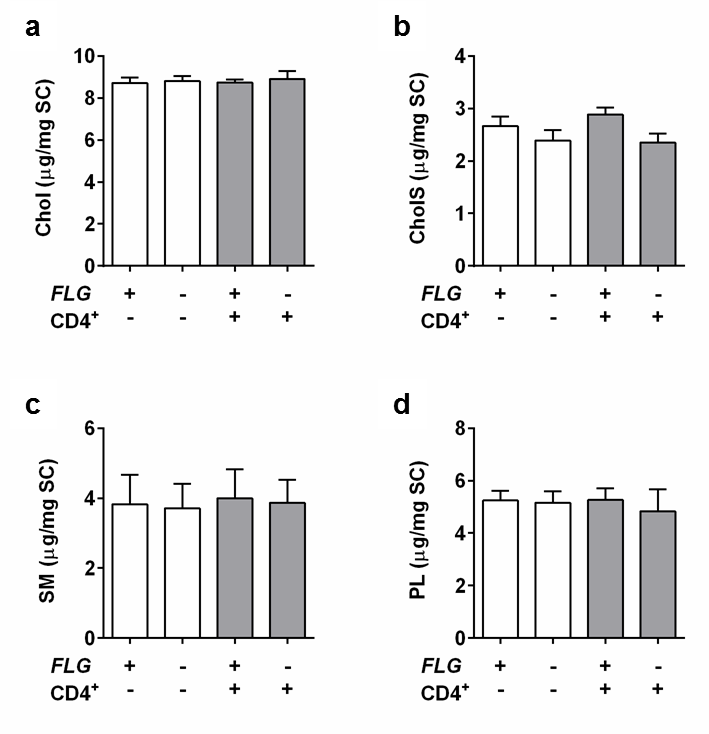


**Figure S5.** High-performance thin layer chromatography analysis of SC lipids
(**a**) cholesterol (Chol), (**b**) cholesteryl sulfate (CholS), (**c**) sphingomyelin (SM) and
(**d**) phospholipids (PL) of *FLG*+ and *FLG*- skin equivalents with or without exposure to activated CD4^+^ T cells (mean ± SEM; n = 4).

**
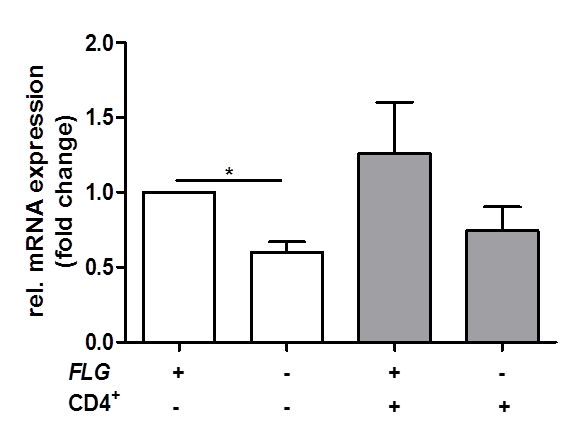
**

**Figure S6.** Relative mRNA expression of *TSLP* in normal (*FLG*+) and filaggrin-deficient (*FLG*-) skin equivalents alone and after addition of activated CD4^+^ T cells (mean ± SEM, n = 4), * indicates statistically significant differences between *FLG*+ skin equivalents (**p* ≤ 0.05).


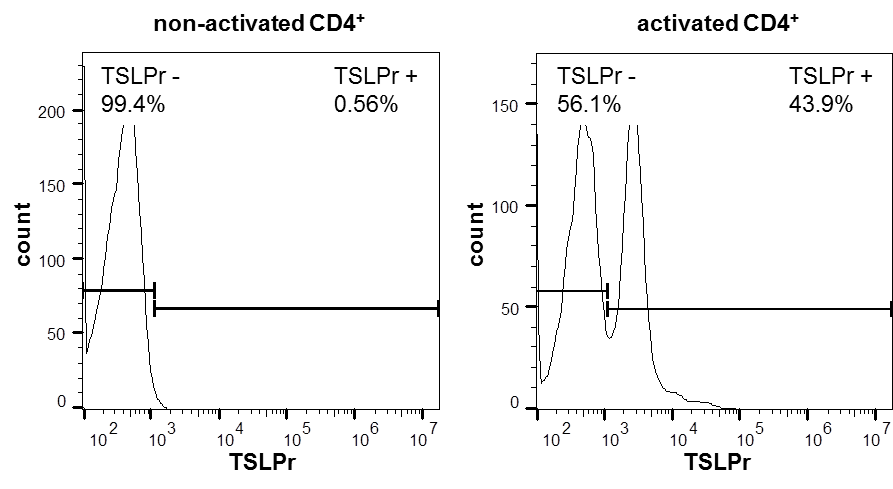


**Figure S7.** TSLP receptor (TSLPr) expression on non-activated and activated CD4^+^ T cells analysed by flow cytometry. After activation of CD4^+^ T cells, TSLPr expression increased significantly from 0.56% to 43.9%.
